# CT-Based Radiomics Analysis for Preoperative Diagnosis of Pancreatic Mucinous Cystic Neoplasm and Atypical Serous Cystadenomas

**DOI:** 10.3389/fonc.2021.621520

**Published:** 2021-06-11

**Authors:** Tiansong Xie, Xuanyi Wang, Zehua Zhang, Zhengrong Zhou

**Affiliations:** ^1^ Department of Radiology, Shanghai Cancer Center, Fudan University, Shanghai, China; ^2^ Department of Oncology, Shanghai Medical College of Fudan University, Shanghai, China; ^3^ Department of Radiation Oncology, Shanghai Cancer Center, Fudan University, Shanghai, China; ^4^ Minhang Branch, Shanghai Cancer Center, Fudan University, Shanghai, China

**Keywords:** pancreas, cystademoma, radiomic analysis, comput(eriz)ed tomography, diagnosis

## Abstract

**Objectives:**

To investigate the value of CT-based radiomics analysis in preoperatively discriminating pancreatic mucinous cystic neoplasms (MCN) and atypical serous cystadenomas (ASCN).

**Methods:**

A total of 103 MCN and 113 ASCN patients who underwent surgery were retrospectively enrolled. A total of 764 radiomics features were extracted from preoperative CT images. The optimal features were selected by Mann-Whitney U test and minimum redundancy and maximum relevance method. The radiomics score (Rad-score) was then built using random forest algorithm. Radiological/clinical features were also assessed for each patient. Multivariable logistic regression was used to construct a radiological model. The performance of the Rad-score and the radiological model was evaluated using 10-fold cross-validation for area under the curve (AUC), sensitivity, specificity, positive predictive value (PPV), negative predictive value (NPV) and accuracy.

**Results:**

Ten screened optimal features were identified and the Rad-score was then built based on them. The radiological model was built based on four radiological/clinical factors. In the 10-fold cross-validation, the Rad-score was proved to be robust and reliable (average AUC: 0.784, sensitivity: 0.847, specificity: 0.745, PPV: 0.767, NPV: 0.849, accuracy: 0.793). The radiological model performed slightly less well in classification (average AUC: average AUC: 0.734 sensitivity: 0.748, specificity: 0.705, PPV: 0.732, NPV: 0.798, accuracy: 0.728.

**Conclusions:**

The CT-based radiomics analysis provided promising performance for preoperatively discriminating MCN from ASCN and showed good potential in improving diagnostic power, which may serve as a novel tool for guiding clinical decision-making for these patients.

## Introduction

Due to the widespread use and development of cross-sectional techniques, pancreatic cystic lesions (PCL) have increasingly been accidentally discovered. According to previous reports, the prevalence of occasionally detected PCL is between 3% and 14% (1). Among PCL, pancreatic cystic neoplasms encompass a diverse group of histopathological entities with different biological behaviors (2). Serous cystadenomas (SCN) represent approximately 33% of PCL, which is now considered a mostly benign cystic neoplasm in the pancreas (3). Most patients with SCN do not need surgery unless they have noticeable symptoms or preoperative diagnosis is unclear. However, in a large sample size study (4), 61% of SCN patients received surgery, and 60% of these surgeries were prompted by an unclear diagnosis. Mucinous cystic neoplasm (MCN) is a easily misdiagnosed lesion of SCN, which is characterized by columnar mucin-producing epithelium supported by ovarian-type stroma (5). Given the risk of malignant transformation, all MCN should be surgically resected once detected. Therefore, differentiating SCN from MCN is clinically critical, and radiology plays a pivotal role in this task.

SCN exhibits variable macroscopic patterns (6, 7). In its most classic imaging appearance, SCN manifests as a microcystic mass with central sunburst-like calcification. This classic radiological pattern comprises 70% of SCN and can be easily differentiated from MCN. However, SCN with atypica l morphological patterns, such as the oligocystic or unilocular variants, has a similar radiological appearance with MCN, making preoperative differentiation difficult (8). Although previous studies have demonstrated that some radiological features could help differentiate atypical SCN (ASCN) and MCN (9–11), preoperative diagnosis remains challenging. Thus, radiologists often report “pancreatic cystadenomas” without highlighting serous or mucinous in the clinical routine. As a result, clinicians apply an aggressive treatment strategy and a significant number of patients with ASCN undergo unnecessary surgery. Furthermore, endoscopic ultrasonography helped in improving diagnostic accuracy with biopsy or cyst fluid analysis guided by it, but its widespread use is difficult to achieve in clinical settings due to invasiveness and complex procedures (12, 13). Therefore, it would be of great utility to develop an accurate, non-invasive, and convenient diagnostic tool for ASCN and MCN patients.

Radiomics, an emerging field first introduced in 2012, extracts a large number of quantitative features from medical imaging and constructs associations between such features and tumor heterogeneity (14). Nowadays, radiomics analysis based on large image datasets has been widely studied in many aspects of oncology (15). Some recently published studies have reported that radiomics is valuable in discriminating SCN from MCN (16–19), which provides a new prospect for resolving this clinical issue. However, the sample size of the above studies is relatively small and lack of validation, which may limit their clinical transformation. In this study, we propose and validate a CT-based radiomics score (Rad-score) to discriminate between ASCN and MCN. We would compare the Rad-score to existing clinical and radiological features in diagnostic performance.

## Materials and methods

### Study Population

This was a single-center study with patients retrospectively enrolled from Fudan University Shanghai Cancer Center. The ethics committee approved the study in our center and the informed consent requirement was waived. A total of 216 patients who underwent surgery between January 2014 and August 2020 were consecutively admitted. These included 103 patients with MCN and 113 patients with ASCN (including oligocystic SCN and unilocular cystic SCN). Oligocystic SCN was defined as SCN with few cysts (< 6) and a single-cyst size larger than 2 cm. Unilocular cystic SCN was defined as SCN containing a single cyst of any macroscopic size. Detailed inclusion and exclusion criteria are listed in the Supplementary Materials.

### CT Protocol

Patients underwent CT scans on multiple CT scanners (SOMATOM Sensation64, SOMATOM Sensation40, SOMATOM Definition AS, Siemens AG Medical Solutions; Brilliance 64, Philips). The acquisition protocol satisfied the following requirements: tube voltage: 120 KV; tube current: 90–270 mA; matrix: 256 × 256; thickness of reconstructed images: 1 mm; three-phases scan; after plain scanning, two-phase contrast-enhanced CT scans were initiated at 30 s (arterial phase), 80 s (venous phase) after injection of the contrast agent. With a high-pressure injector, 100 ml of a nonionic-contrast agent was injected at a rate of 2.5 ml/s.

Preoperative CT scans were recorded and extracted from the picture archiving and communication system in the Department of Radiology at Fudan University Shanghai Cancer Center. The venous-phase CT digital imaging and communication in medicine images were required and then loaded into a personal computer for further radiomics analysis.

### Radiomics Analysis and Radiomics Score Building

**Figure 1:**
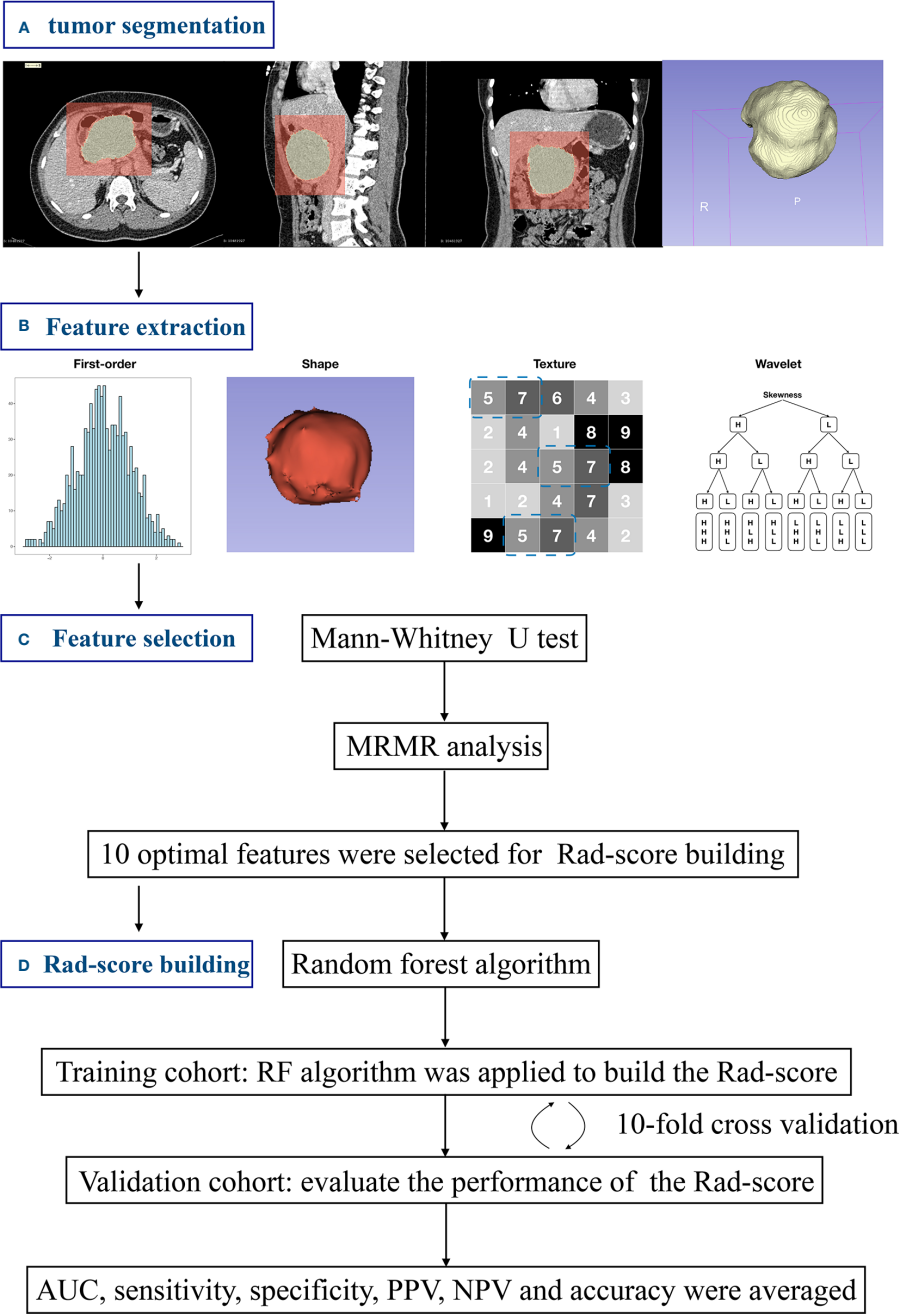
Workflow of the radiomics analysis. **(A)** Tumors were semi-manually segmented on all slices. **(B)** Radiomics features were extracted. **(C)** Feature selection procedure was used to identity the optimal feature set. **(D)** The Rad-score was built using random forest method and validated by 10-fold cross validation.

The workflow of radiomics analysis is illustrated in **Figure 1**. Image preprocessing and tumor segmentation were performed *via* 3D Slicer software (20) (version 4.11.0; http://www.slicer.prg). Feature extraction was performed based on Pyradiomics package (version 2.2.0; https://pyradiomics.readthedocs.io/) (21). A total of 764 radiomics features were extracted based on venous-phase CT images (**Table 1**). Thirty patients were randomly chosen to evaluate the inter/intra-observer intraclass correlation coefficient (ICC) of radiomics features. Features with both inter-observer and intra-observer ICC higher than 0.90 were allowed for feature engineering. The processes of image preprocessing, segmentation, feature extraction and reproducibility analysis are provided in the Supplementary Materials.

**Table 1:** List of Radiomics feature classes.

Radiomics feature class	Description
Shape	Descriptors of the 3D/2D-size and shape of the ROI
First-order	Describe the distribution of voxel intensities within the ROI
GLDM	Quantify the gray level dependencies (the number of connected voxels within a certain distance that are dependent on the center voxel) in the ROI
GLRLM	Quantify the gray level runs (the length in number of voxels that have the same intensity)
GLSZM	Quantify gray level zones (the number of connected voxels with the same intensity) in the ROI
Wavelet-based	Wavelet transformation based on above features

*GLDM*, gray level dependence matrix; *GLRLM*, gray level run length matrix; *GLSZM*, gray level size zone matrix.

A univariable analysis was performed using the Mann–Whitney U test to compare features between MCN and ASCN. Given testing of multiple comparisons, p-values are adjusted for false discovery rate using Benjamini-Hochberg method (22). With false discovery rate set at 5%, radiomics features exhibiting significant difference were kept for further analysis. With the remaining features, the minimum redundancy maximum relevance (MRMR) method was applied to remove redundant unrelated features. Finally, 10 screened optimal features were identified by MRMR method. The Rad-score was then built using random forest method based on optimal features.

### Radiological Analysis and Radiological Model Building

Two junior radiologists independently reviewed all CT images and evaluated radiological features. They were unaware of the pathological diagnosis. The following radiological features were assessed for each patient: (i) tumor size; (ii) location; (iii) lesion contour (round/ovoid, or lobulated); (iv) wall thickness; (v) wall enhancement; (vi) calcification; (vii) mural nodule; (viii) dilation of the Wirsung duct. Diagnostic criteria and examples are detailed in the Supplementary Materials. In cases of disagreement, a third senior radiologist would assess and draw a final conclusion. Clinical characteristics—including age, gender, chief complaint, serum carbohydrate antigen 19-9 (CA19-9) level, serum carcinoma embryonic antigen (CEA) level, and serum carbohydrate antigen 125 (CA125) level—were derived from the patients’ medical records.

Stepwise logistic regression was used to identify powerful factors and construct a radiological model. A univariate analysis was conducted to compare the differences between MCN and ASCN in radiological features and clinical characteristics. Significant factors with p < 0.05 in the univariate analysis were subsequently introduced into a multivariate logistic regression analysis. A forward stepwise factor selection was performed using the likelihood ratio test and Akaike information criterion (AIC).

### Model Validation and Assessment

In order to avoid over-optimized estimation, 10-fold cross validation was applied to assess both the Rad-score and the radiological model. The 10-fold cross validation has been a commonly used method in previously reported studies to avoid confounders arisen from single data assignment (23–25). In the 10-fold cross validation, the patients were randomly allocated to training and validation sets in a 9:1 ratio for 10 times. During each-time validation, the training set was used to train a new model. The validation set was used to evaluate the performance of the trained model. After 10-time validation, average area under the receiver operating parameters (AUC), sensitivity, specificity, positive predictive value (PPV), negative predictive value (NPV) and accuracy in the validation set were calculated to assess the model.

All statistical analyses were performed using R software (R, version 4.0.2, http://www.r-project.org). A two-sided p-value < 0.05 was indicative of a statistically significant difference. The R packages used in this study are reported in the Supplementary Materials.

## Results

### Study Population

The clinical and radiological characteristics of the patients are summarized in **Table 2**. The mean age of ASCN patients was 46.16 ± 11.82 years, and that of MCN patients was 44.80 ± 13.31 years. There was no significant difference in age between the two groups (p = 0.426). There were 21 males and 92 females in the ASCN group. However, in the MCN group, except for six male patients, the remaining 97 patients were all female. The difference between patients’ gender was significant (p = 0.005). Among the serum tumor markers, only CA19-9 demonstrated a significant difference between the two groups, but the absolute difference in its median value was only 3.48 U/ml (p = 0.003).

**Table 2:** Clinical and radiological characteristics of pancreatic mucinous cystic neoplasm and atypical serous cystadenoma.

Variables	Atypical serous cystadenoma	Mucinous cystic neoplasm	*p*-value
Ages (years)	46.16±11.82	44.80±13.31	0.426
Sex			
Male	21 (18.6%)	6 (5.8%)	0.005
Female	92 (81.4%)	97 (94.2%)	
Symptoms			0.467
Negative	84 (74.3%)	72 (69.9%)	
Positive	29 (25.7%)	31 (30.1%)	
Tumor marker			
CA19-9 (U/ml)	9.02 (6.25-14.44)	12.50 (7.33-22.50)	0.003
CEA (ng/ml)	1.56 (0.93-1.96)	1.44 (0.88-2.27)	0.766
CA125 (U/ml)	11.37 (8.41-17.60)	12.00 (9.22-19.47)	0.112
Size	3.00 (2.10-3.85)	4.00 (2.90-5.70)	<0.001
Location			<0.001
Head/Neck	54 (47.8%)	15 (14.6%)	
Body/Tail	59 (52.2%)	88 (85.4%)	
Leison contour			0.186
Round/Ovoid	74 (65.5%)	76 (73.8%)	
Lobulated	39 (34.5%)	27 (26.2%)	
Wall thicknes			0.043
Thin	102 (90.3%)	83 (80.6%)	
Thick	11 (9.7%)	20 (19.4%)	
Wall enhancement			<0.001
Negative	81 (71.7%)	47 (45.6%)	
Positive	32 (28.3%)	56 (54.4%)	
Calcification			0.115
Negative	98 (86.7%)	81 (78.6%)	
Positive	15 (13.3%)	22 (21.4%)	
Mural nodules			0.531
Negative	99 (87.6%)	93 (90.3%)	
Positive	14 (12.4%)	10 (9.7%)	
Dilation of the Wirsung duct			0.277
Negative	109 (96.5%)	96 (93.2%)	
Positive	4 (3.5%)	7 (6.8%)	

Chi-Square tests were used to compare the difference in categorical variables (sex, symptoms, location, lesion contour, wall thickness, wall enhancement, calcification, mural nodules, and dilation of the Wirsung duct). A two-sample t-test was used to compare the difference in age. A Mann-Whiney U test was used to compare the difference in serum tumor makers and tumor size. CA19-9, carbohydrate antigen 19-9; CEA, carcinoma embryonic antigen; CA125, carbohydrate antigen 125.

### Rad-Score Building and Validation

**Figure 2:**
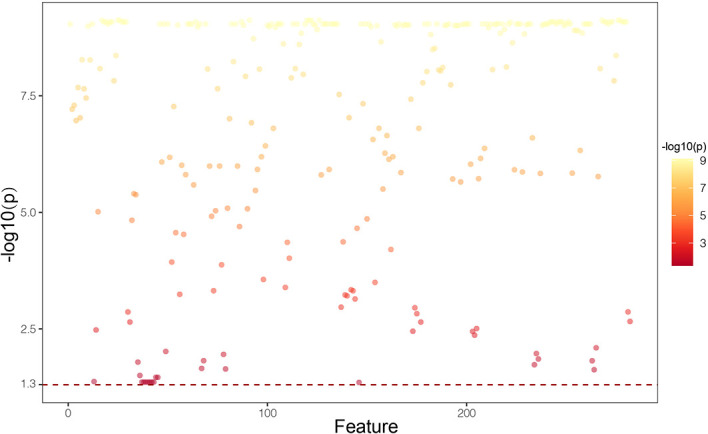
Manthattan plot showing p-values of 282 radiomics features that exhibited significant difference between mucinous cystic neoplasm and atypical serous cystadenomas. P-values are adjusted for false discovery rate using Benjamini-Hochberg method.

**Figure 3:**
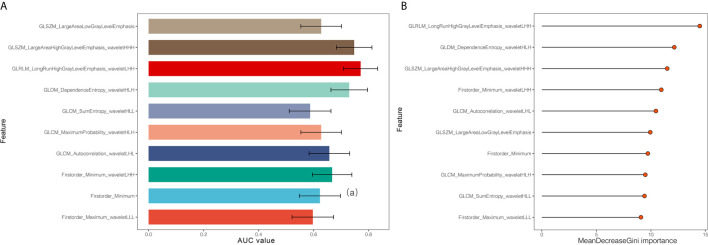
**(A)** Area under the curve of 10 optimal radiomics features identified by minimum redundancy maximum relevance method. **(B)** Display of importance of 10 optimal radiomics features in random forest classifier built in the full dataset.

**Figure 4:**
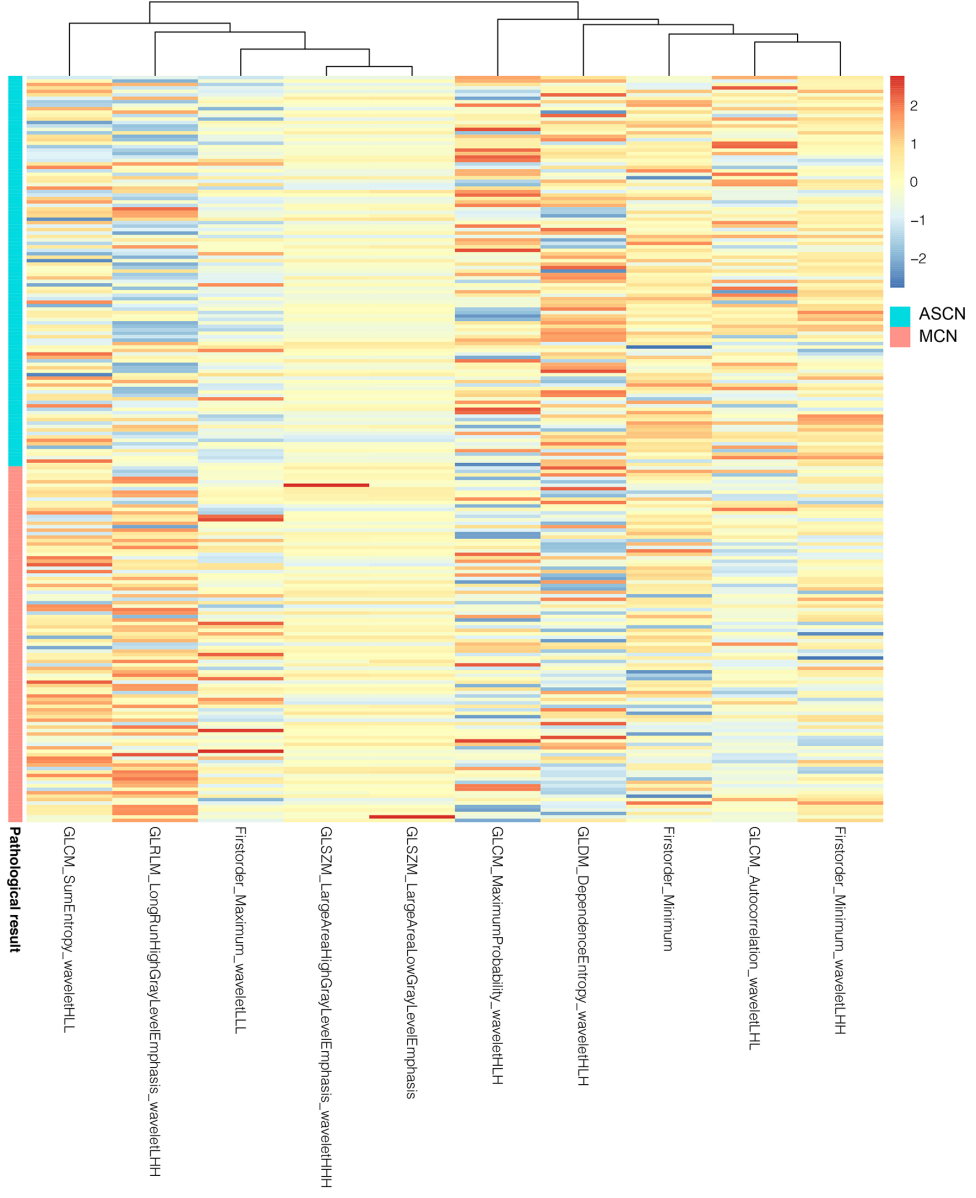
Heatmap of 10 optimal radiomics features of 216 enrolled patients. The radiomics features were normalized according to Z-score.

In the reproducibility analysis, there were 472 radiomics features with both inter-observer ICC and intra-observer ICC higher than 0.90. With a Mann–Whitney U test and a (Benjamini-Hochberg adjustment) false discovery rate of 5%, 282 radiomics features were reserved. The adjusted p-values of these features are shown in **Figure 2**. Finally, 10 screened optimal features were identified by MRMR method (Firstorder_Minimum, GLCM_Sumentropy_waveletHLL, Firstorder_Maximum_waveletLLL, GLDM_DependenceEntropy_waveletHLH, GLCM_MaximumProbability_waveletHLH, GLCM_Autocorrelation_waveletLHL, GLSZM_LargeAreaHighGrayLevelEmphasis_waveletHHH, GLSZM_LargeAreaLowGrayLevelEmphasis, Firstorder_Minimum_waveletLHH, GLRLM_LongRunHighGrayLevelEmphasis_wavelteLHH). The comparisons of 10 optimal features between MCN and ASCN are listed in **Table 3**. The diagnostic performance of each optimal feature was assessed *via* ROC analysis (**Table 4**), and the AUC bar plot of 10 features in discriminating MCN from ASCN was illustrated in **Figure 3A**. **Figure 4** shows the heatmap of 10 optical features in full dataset.

**Table 3:** Comparisons of 10 optimal radiomics features in distinguishing between mucinous cystic neoplasm and atypical serous cystadenomas.

Features	Atypical serous cystadenoma	Mucinous cystic neoplasm	*p*-value
Firstorder_Minimum	0.96 (0.86, 1.08)	0.91 (0.75, 1.03)	0.003
GLCM_SumEntropy_waveletHLL	1.52 (1.50, 1.53)	1.52 (1.51, 1.53)	0.045
Firstorder_Maximum_waveletLLL	4.02 (3.78, 4.35)	4.11 (3.90, 4.60)	0.024
GLDM_DependenceEntropy_waveletHLH	4.60 (4.49, 4.66)	4.47 (4.38, 4.56)	<0.001
GLCM_MaximumProbability_waveletHLH	0.28 (0.28, 0.28)	0.28 (0.27, 0.28)	0.002
GLCM_Autocorrelation_waveletLHL	2.33 (2.28, 2.38)	2.29 (2.28, 2.32)	<0.001
GLSZM_LargeAreaHighGrayLevelEmphasis_waveletHHH	14330720.57 (5489652.56, 43035885.79)	66623017.63 (18474345.51, 225682538.90)	<0.001
GLSZM_LargeAreaLowGrayLevelEmphasis	306285001.00 (71503936.00, 1300000000.00)	911436100.00 (214510204.45, 4578654406.50)	0.001
Firstorder_Minimum_waveletLHH	-0.12 (-0.16, -0.08)	-0.16 (-0.20, -0.12)	<0.001
GLRLM_LongRunHighGrayLevelEmphasis_waveletLHH	13.43 (12.37, 14.26)	14.87 (13.93, 15.91)	<0.001

Data is expressed as median (interquartile range). P-values are adjusted using Benjamini-Hochberg method, with false discovery rate set at 5%.

**Table 4:** Confusion matrix analyses of 10 optimal radiomics features.

Feature	AUC	CI	Cutoff-value	Sensitivity	Specificity	Accuracy	PPV	NPV
Firstorder_Minimum	0.623	0.548-0.698	0.777	0.291	0.929	0.625	0.789	0.59
GLCM_SumEntropy_waveletHLL	0.587	0.511-0.663	1.528	0.330	0.814	0.583	0.618	0.571
Firstorder_Maximum_waveletLLL	0.597	0.522-0.673	3.980	0.487	0.68	0.579	0.547	0.625
GLDM_DependenceEntropy_waveletHLH	0.730	0.663-0.796	4.454	0.466	0.867	0.676	0.762	0.641
GLCM_MaximumProbability_waveletHLH	0.628	0.554-0.702	0.279	0.718	0.487	0.597	0.561	0.655
GLCM_Autocorrelation_waveletLHL	0.657	0.584-0.731	2.331	0.825	0.504	0.657	0.603	0.760
GLSZM_LargeAreaHighGrayLevelEmphasis_waveletHHH	0.748	0.683-0.812	27502194.84	0.718	0.681	0.699	0.673	0.726
GLSZM_LargeAreaLowGrayLevelEmphasis	0.628	0.554-0.702	482639205.8	0.612	0.593	0.602	0.578	0.626
Firstorder_Minimum_waveletLHH	0.668	0.596-0.739	-0.147	0.573	0.690	0.634	0.628	0.639
GLRLM_LongRunHighGrayLevelEmphasis_waveletLHH	0.771	0.709-0.833	14.646	0.563	0.867	0.722	0.795	0.685

AUC, area under the curve; CI, confidence interval; PPV, positive predictive value; NPV, negative predictive value.

**Figure 5:**
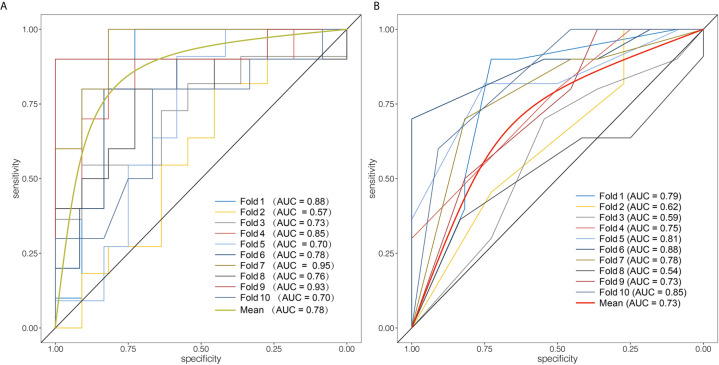
Evaluation of the diagnostic performance of the Rad-score and the radiological model. (**A)** Receiver operating curves of 10-fold cross validation of the Rad-score. The Rad-score achieved average AUC of 0.78. (**B)** Receiver operating curves of 10-fold cross validation of the radiological model. The average AUC of the radiological model was 0.73.

Next, random forest algorithm was applied based on above optimal feature set to build the Rad-score. The mean decrease Gini importance index of each feature is illustrated in **Figure 3B**. In order to avoid over-optimized estimation of the Rad-score, 10-fold cross validation was applied. The average AUC, sensitivity, specificity, PPV, NPV and accuracy of the Rad-score were 0.784, 0.847, 0.745, 0.747, 0.849 and 0.793, respectively, which demonstrated that the Rad-score was a robust and reliable diagnostic tool. The ROC analysis of 10-time cross validation is shown in **Figure 5A**.

### Radiological Model Building and Validation

The radiological features of ASCN and MCN are listed in **Table 2**. In the univariate analysis, four radiological features had a significant association with the pathological results, including size, location, wall thickness, and wall enhancement. However, the absolute median value difference in size between ASCN and MCN was only 1.00 centimeters. From the perspective of clinical practice, the differences in tumor size and serum CA19-9 levels were not pronounced enough to prompt radiologists to make diagnoses, despite their statistical significance. For this reason, they were excluded. Finally, three radiological features, together with one clinical factor (gender), were subjected to multivariate logistic regression. Based on the full dataset, stepwise logistic regression was applied. After the forward stepwise factor selection, three factors (location, wall enhancement and gender) were introduced to construct the radiological model. **Table 5** listed the detailed parameters of the radiological model.

**Table 5:** Parameters of the radiological model built in the full dataset.

Variables	Coefficient	Odds ratio	95% CI	*p*-value	Akaike information criteria
Intercept	-2.479			< 0.001	262.379
Location	1.558	4.749	2.391-9.432	< 0.001	
Wall enhancement	1.030	2.802	1.529-5.133	< 0.001	
Gender	0.968	2.632	0.942-7.356	0.065	

CI, confidence interval.

Subsequently, 10-fold cross validation was used to assess the performance of the radiological model. ROC analysis in 10-fold cross validation revealed a slightly worse performance of the radiological model than that of the Rad-score (average AUC: 0.734, sensitivity: 0.748, specificity: 0.705, PPV: 0.732, NPV: 0.798, accuracy: 0.728). **Figure 5B** shows the ROC analysis of the radiological model in 10-time cross validation.

## Discussion

Preoperative distinguishing between MCN and ASCN remains a clinical dilemma. Our study investigated and validated the value of the CT based Rad-score in preoperatively discriminating MCN and ASCN. We identified optimal radiomics feature set in classification and built the Rad-score by random forest method. The 10-fold cross validation indicated that the Rad-score was a robust and stable imaging biomarker to discriminate between MCN and ASCN. Furthermore, a radiological model was also constructed based on radiological and clinical factors. Our results demonstrated that the Rad-score exhibited better diagnostic performance than the radiological model in 10-fold cross validation. To the best of our knowledge, this is the first study investigating the value of radiomics analysis in discriminating MCN and ASCN with comparison to radiological analysis.

Given the difference in biological behavior and treatment principle, preoperative differentiation between MCN and SCN is critical to developing a treatment strategy. Although ASCN is relatively uncommon, Kang et al. (26) demonstrated that the preoperative differential diagnosis of ASCN and MCN is more difficult than that of typical SCN. Thus, a new diagnostic method was needed to resolve this clinical problem. Among the clinical characteristics, gender and lesion location have symbolic significance for diagnosis because MCN, unlike ASCN, occurs almost exclusively in females and pancreatic bodies or tails (27). Therefore, for male patients with cystic lesion located in the pancreatic head, the diagnosis of MCN should be made cautiously. Some researchers have supposed that MCN is related to female hormones (28). The gender distribution and characteristic ovarian-type stroma of MCN might support this hypothesis, but it could not totally explain why a few MCN occurred in males (29). From a radiological perspective, in 2003, the study by Frank et al. (9) first investigated what CT signs are valuable to distinguish ASCN from MCN. Location, lesion contour, and wall enhancement were specific for differential diagnosis in their study, which is part consistent with our conclusion. After that, Kim et al. (10) also reported that lesion contour was of great significance to differentiate ASCN and MCN, but the location parameter was not statistically significant, which might be attributed to the small sample size of that study. Furthermore, the presence and location of calcification helped differentiate SCN from MCN (7). However, regarding to ASCN and MCN, the value of calcification remains unclear. Our study found that there was no significant difference between the two groups in the presence of calcification (p = 0.115). In terms of serum tumor makers, Bassi et al. (30) proposed that CEA, CA19-9, and CA125 might be valuable in the preoperative diagnosis of SCN and MCN. Nevertheless, our study showed that these serum tumor makers were of limited value, with only serum CA19-9 levels showing a statistical difference between the two groups, but little absolute difference.

Radiomics is promising to solve this clinical dilemma. As a novel image analysis method, radiomics have shown great potential for tumor classification and discrimination in organs, such as the lung, liver, and kidney (31–33). Radiomics features extracted from the medical images might capture histopathological heterogeneity. Different pathological types of tumors exhibit different values of radiomics features, which might be an underlying mechanism of applying radiomics in tumor classification. Several recent studies have investigated the value of radiomics in differentiating SCN from MCN. Shen et al. (19) applied CT radiomics analysis to differentiate SCN, MCN and intraductal papillary mucinous neoplasms using three different machine learning algorithms. They found that the random forest classifier achieved highest accuracy. The study of Yang et al. (17) provided preliminary results suggesting that CT textural features helped in differentiate SCN and MCN. The AUC of textural features was in the range of 0.7–0.8, which is slightly higher than that of optimal features in our study. Furthermore, this team found that combining radiological characteristics and texture analysis could achieve higher diagnostic performance (18). It is worth noting that the most valuable feature (LongRunHighGrayLevelEmphasis) in our study was also selected by Yang et al., although it was transformed by wavelet transformation in our study. This discrepancy might be attributed to different radiomics software, feature selection methods and inclusion criteria were applied in the two studies. As a result, we speculated that this feature is stable and worthy of further investigation. Furthermore, an additional study applied radiomics to differentiate ASCN and MCN and compared radiomics and radiological analysis (16). It demonstrated that adding radiological features into radiomics model could significantly improve the model’s calibration performance. These studies suggest that radiomics is a promising means of discriminating ASCN from MCN, but most of these studies had limited sample size and lacked of comparison to existing radiological analysis. In the present study, we constructed a CT based Rad-score and performed 10-fold cross validation to prove the robustness of it. Further analysis revealed that the Rad-score provided better discrimination performance in distinguishing MCN from ASCN than that of radiological model.

The present study has some limitations. First, it is a single-center retrospective study. The validity of our conclusions still needs to be further explored and confirmed. Second, the pathological implications of the Rad-score remain unclear. For a better understanding and acceptance of radiomics, further studies are warranted to confirm the hypothesis that radiomics reflects tumor heterogeneity. Third, only CT images were analyzed in our study. Multi-modal imaging method needs to be further explored to improve diagnostic accuracy in the future.

In conclusion and concordance with previous studies, the Rad-score based on CT might serve as a novel image biomarker to preoperatively discriminate ASCN from MCN. CT radiomics analysis show promising ability to improve diagnostic accuracy, which is expected to optimize the treatment regimen and avoid unnecessary surgery. Further studies are needed to confirm the robustness of our conclusions.

## Data Availability Statement

The datasets presented in this article are not readily available because the images and clinical data are related to patients’ privacy in our center. Requests to access the datasets should be directed to zhouzr-16@163.com.

## Ethics Statement

The studies involving human participants were reviewed and approved by the ethics committee. They approved the study in Fudan University Shanghai Cancer Center and the informed consent requirement was waived. The ethics committee waived the requirement of written informed consent for participation.

## Author Contributions

TSX and XYW contributed equally to this work. TSX and XYW designed the study, performed the data collection and analysis, and drafted the manuscript. ZHZ collected the data. ZRZ designed the study and modified the manuscript. All authors contributed to the article and approved the submitted version.

## Funding

The study was supported by funds from the Scientific and Innovative Plan of Shanghai (18140901200), National Major Science and Technology Projects of China (2020ZX09201-013), Suzhou Health Professionals Scientific Project (GSWS2019020) and Natural Science Science Fund of Jiangsu Province (BK20181179).

## Conflict of Interest

The authors declare that the research was conducted in the absence of any commercial or financial relationships that could be construed as a potential conflict of interest.

## References

[1] Hasan A Visrodia K Farrell JJ Gonda TA. Overview and Comparison of Guidelines for Management of Pancreatic Cystic Neoplasms. World J Gastroenterol (2019) 2531:4405–13. 10.3748/wjg.v25.i31.4405 PMC6710181 31496620

[2] van Huijgevoort NCM Del Chiaro M Wolfgang CL van Hooft JE Besselink MG. Diagnosis and Management of Pancreatic Cystic Neoplasms: Current Evidence and Guidelines. Nat Rev Gastroenterol Hepatol (2019) 1611:676–89. 10.1038/s41575-019-0195-x 31527862

[3] Reid MD Choi H Balci S Akkas G Adsay V. Serous Cystic Neoplasms of the Pancreas: Clinicopathologic and Molecular Characteristics. Semin Diagn Pathol (2014) 316:475–83. 10.1053/j.semdp.2014.08.009 25441309

[4] Jais B Rebours V Malleo G Salvia R Fontana M Maggino L. Serous Cystic Neoplasm of the Pancreas: A Multinational Study of 2622 Patients Under the Auspices of the International Association of Pancreatology and European Pancreatic Club (European Study Group on Cystic Tumors of the Pancreas). Gut (2016) 652:305–12. 10.1136/gutjnl-2015-309638 26045140

[5] Fernández-del Castillo C. Mucinous Cystic Neoplasms. J Gastrointestinal surgery: Off J Soc Surg Alimentary Tract (2008) 123:411–3. 10.1007/s11605-007-0347-0 17955316

[6] Choi JY Kim MJ Lee JY Lim JS Chung JJ Kim KW. Typical and Atypical Manifestations of Serous Cystadenoma of the Pancreas: Imaging Findings With Pathologic Correlation. AJR Am J Roentgenol (2009) 1931:136–42. 10.2214/AJR.08.1309 19542405

[7] Chu LC Singhi AD Haroun RR Hruban RH Fishman EK. The Many Faces of Pancreatic Serous Cystadenoma: Radiologic and Pathologic Correlation. Diagn Interv Imaging (2017) 983:191–202. 10.1016/j.diii.2016.08.005 27614585

[8] Goh BK Tan YM Yap WM Cheow PC Chow PK Chung YF. Pancreatic Serous Oligocystic Adenomas: Clinicopathologic Features and a Comparison With Serous Microcystic Adenomas and Mucinous Cystic Neoplasms. World J Surg (2006) 308:1553–9. 10.1007/s00268-005-0749-7 16773248

[9] Cohen-Scali F Vilgrain V Brancatelli G Hammel P Vullierme MP Sauvanet A. Discrimination of Unilocular Macrocystic Serous Cystadenoma From Pancreatic Pseudocyst and Mucinous Cystadenoma With CT: Initial Observations. Radiology (2003) 2283:727–33. 10.1148/radiol.2283020973 12954892

[10] Kim SY Lee JM Kim SH Shin KS Kim YJ An SK. Macrocystic Neoplasms of the Pancreas: CT Differentiation of Serous Oligocystic Adenoma From Mucinous Cystadenoma and Intraductal Papillary Mucinous Tumor. AJR Am J Roentgenol (2006) 1875:1192–8. 10.2214/AJR.05.0337 17056905

[11] Lin XZ Wu ZY Li WX Zhang J Xu XQ Chen KM. Differential Diagnosis of Pancreatic Serous Oligocystic Adenoma and Mucinous Cystic Neoplasm With Spectral CT Imaging: Initial Results. Clin Radiol (2014) 6910:1004–10. 10.1016/j.crad.2014.05.003 24919983

[12] Sydney GI Ioakim KJ Michaelides C Sepsa A Sopaki-Valalaki A Tsiotos GG. EUS-FNA Diagnosis of Pancreatic Serous Cystadenoma With the Aid of Cell Blocks and Alpha-Inhibin Immunochemistry: A Case Series. Diagn Cytopathol (2020) 483:239–43. 10.1002/dc.24348 31785091

[13] Faias S Pereira L Roque R Chaves P Torres J Cravo M. Excellent Accuracy of Glucose Level in Cystic Fluid for Diagnosis of Pancreatic Mucinous Cysts. Digestive Dis Sci (2020) 657:2071–8. 10.1007/s10620-019-05936-5 31705344

[14] Lambin P Rios-Velazquez E Leijenaar R Carvalho S van Stiphout RG Granton P. Radiomics: Extracting More Information From Medical Images Using Advanced Feature Analysis. Eur J Cancer (2012) 484:441–6. 10.1016/j.ejca.2011.11.036 PMC4533986 22257792

[15] Rogers W Thulasi Seetha S Refaee TAG Lieverse RIY Granzier RWY Ibrahim A. Radiomics: From Qualitative to Quantitative Imaging. Br J Radiol (2020) 931108:20190948. 10.1259/bjr.20190948 PMC7362913 32101448

[16] Xie H Ma S Guo X Zhang X Wang X. Preoperative Differentiation of Pancreatic Mucinous Cystic Neoplasm From Macrocystic Serous Cystic Adenoma Using Radiomics: Preliminary Findings and Comparison With Radiological Model. Eur J Radiol (2020) 122:108747. 10.1016/j.ejrad.2019.108747 31760275

[17] Yang J Guo X Ou X Zhang W Ma X. Discrimination of Pancreatic Serous Cystadenomas From Mucinous Cystadenomas With CT Textural Features: Based on Machine Learning. Front Oncol (2019) 9:494. 10.3389/fonc.2019.00494 31245294 PMC6581751

[18] Yang J Guo X Zhang H Zhang W Song J Xu H. Differential Diagnosis of Pancreatic Serous Cystadenoma and Mucinous Cystadenoma: Utility of Textural Features in Combination With Morphological Characteristics. BMC Cancer (2019) 191:1223. 10.1186/s12885-019-6421-7 PMC6915993 31842793

[19] Shen X Yang F Yang P Yang M Xu L Zhuo J. A Contrast-Enhanced Computed Tomography Based Radiomics Approach for Preoperative Differentiation of Pancreatic Cystic Neoplasm Subtypes: A Feasibility Study. Front Oncol (2020) 10:248. 10.3389/fonc.2020.00248 32185129 PMC7058789

[20] Fedorov A Beichel R Kalpathy-Cramer J Finet J Fillion-Robin JC Pujol S. 3D Slicer as an Image Computing Platform for the Quantitative Imaging Network. Magnetic Resonance Imaging (2012) 309:1323–41. 10.1016/j.mri.2012.05.001 PMC3466397 22770690

[21] van Griethuysen JJM Fedorov A Parmar C Hosny A Aucoin N Narayan V. Computational Radiomics System to Decode the Radiographic Phenotype. Cancer Res (2017) 7721:E104–7. 10.1158/0008-5472.Can-17-0339 PMC5672828 29092951

[22] Guo WG Rao MB. On Optimality of the Benjamini-Hochberg Procedure for the False Discovery Rate. Stat Probab Lett (2008) 7814:2024–30. 10.1016/j.spl.2008.01.069

[23] Ren S Zhao R Cui W Qiu W Guo K Cao Y. Computed Tomography-Based Radiomics Signature for the Preoperative Differentiation of Pancreatic Adenosquamous Carcinoma From Pancreatic Ductal Adenocarcinoma. Front Oncol (2020) 10:1618. 10.3389/fonc.2020.01618 32984030 PMC7477956

[24] Liu Z Li M Zuo C Yang Z Yang X Ren S. Radiomics Model of Dual-Time 2-[(18)F]FDG PET/CT Imaging to Distinguish Between Pancreatic Ductal Adenocarcinoma and Autoimmune Pancreatitis. Eur Radiol (2021). 10.1007/s00330-021-07778-0 33677645

[25] Fields BKK Demirjian NL Hwang DH Varghese BA Cen SY Lei X. Whole-Tumor 3D Volumetric MRI-Based Radiomics Approach for Distinguishing Between Benign and Malignant Soft Tissue Tumors. Eur Radiol (2021). 10.1007/s00330-021-07914-w 33893534

[26] Kang JS Kim HJ Choi YJ Byun Y Lee JM Han Y. Clinicoradiological Features of Resected Serous Cystic Neoplasms According to Morphological Subtype and Preoperative Tentative Diagnosis: Can Radiological Characteristics Distinguish Serous Cystic Neoplasms From Other Lesions? Ann Surg Treat Res (2020) 985:247–53. 10.4174/astr.2020.98.5.247 PMC7200608 32411629

[27] Crippa S Fernández-Del Castillo C Salvia R Finkelstein D Bassi C Domínguez I. Mucin-Producing Neoplasms of the Pancreas: An Analysis of Distinguishing Clinical and Epidemiologic Characteristics. Clin Gastroenterol Hepatol (2010) 82:213–9. 10.1016/j.cgh.2009.10.001 PMC3135334 19835989

[28] Regi P Salvia R Cena C Girelli R Frigerio I Bassi C. Cystic “Feminine” Pancreatic Neoplasms in Men. do Any Clinical Alterations Correlate With These Uncommon Entities? Int J Surg (London England) (2013) 112:157–60. 10.1016/j.ijsu.2012.12.008 23274554

[29] Ethun CG Postlewait LM McInnis MR Merchant N Parikh A Idrees K. The Diagnosis of Pancreatic Mucinous Cystic Neoplasm and Associated Adenocarcinoma in Males: An Eight-Institution Study of 349 Patients Over 15 Years. J Surg Oncol (2017) 1157:784–7. 10.1002/jso.24582 PMC5560255 28211072

[30] Bassi C Salvia R Gumbs AA Butturini G Falconi M Pederzoli P. The Value of Standard Serum Tumor Markers in Differentiating Mucinous From Serous Cystic Tumors of the Pancreas: CEA, Ca 19-9, Ca 125, Ca 15-3. Langenbecks Arch Surg (2002) 3877-8:281–5. 10.1007/s00423-002-0324-8 12447553

[31] Fan L Fang M Li Z Tu W Wang S Chen W. Radiomics Signature: A Biomarker for the Preoperative Discrimination of Lung Invasive Adenocarcinoma Manifesting as a Ground-Glass Nodule. Eur Radiol (2019) 292:889–97. 10.1007/s00330-018-5530-z 29967956

[32] Mokrane FZ Lu L Vavasseur A Otal P Peron JM Luk L. Radiomics Machine-Learning Signature for Diagnosis of Hepatocellular Carcinoma in Cirrhotic Patients With Indeterminate Liver Nodules. Eur Radiol (2020) 301:558–70. 10.1007/s00330-019-06347-w 31444598

[33] Huang ZX Li M He D Wei Y Yu HP Wang Y. Two-Dimensional Texture Analysis Based on CT Images to Differentiate Pancreatic Lymphoma and Pancreatic Adenocarcinoma: A Preliminary Study. Acad Radiol (2019) 268:E189–95. 10.1016/j.acra.2018.07.021 30193819

