# Supplementary material for: CT-Based Radiomics Analysis for Preoperative Diagnosis of Pancreatic Mucinous Cystic Neoplasm and Atypical Serous Cystadenomas
Source: Front Oncol. 2021 Jun 11;11:621520. doi: 10.3389/fonc.2021.621520 (PMC8231011; doi:10.3389/fonc.2021.621520)
Supplement: Supplementary file 1 [file DataSheet_1.pdf]

Supplementary materials

1. Inclusion and exclusion criteria

The inclusion criteria were as follows:

(i) Surgery was performed at our center, and the pathological diagnosis was made;

(ii) Preoperative abdominal CT examination was conducted within two weeks before surgery;

(iii) With complete clinicopathological data in electronic medical records.

The exclusion criteria were as follows:

(i) Preoperative CT was not performed at our center;

(ii) Artifact was present in CT images;

(iii) With incomplete CT scanning series;

(iv) MCN that had progressed to invasive carcinoma.

The flow diagram is illustrated in Fig S1.

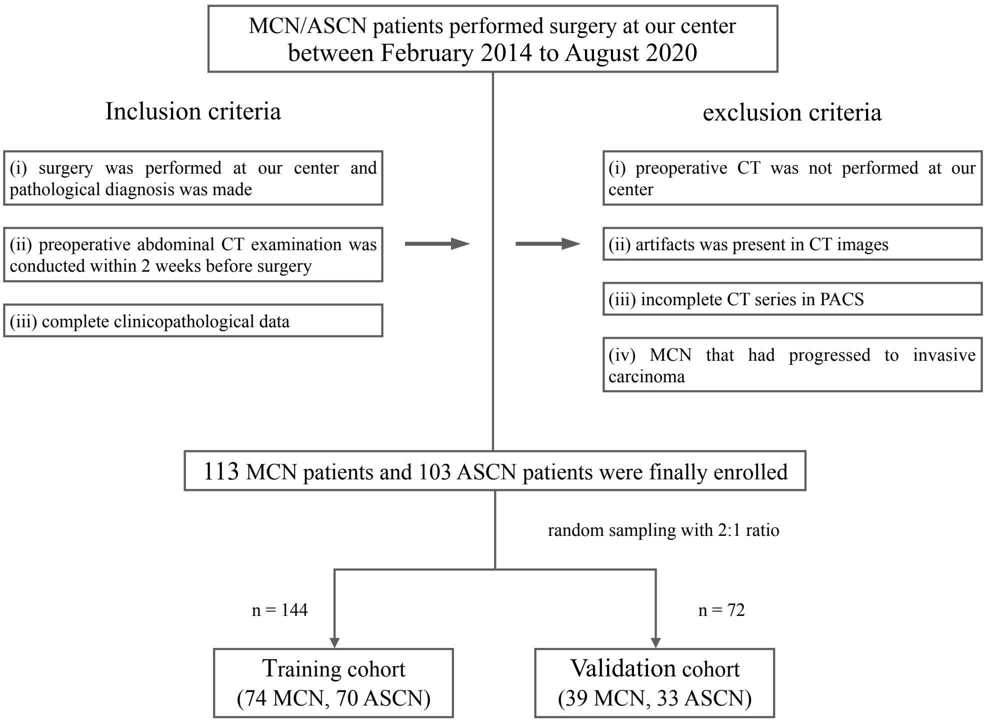

Fig S1. Flow diagram of the inclusion and exclusion processes

2. Image preprocessing, tumor segmentation and feature extraction

Tumor segmentation and image preprocessing were performed via 3D Slicer [1] (version 4.11.0; http://www.slicer.prg). All cases were semi-manually segmented by reader 1 (T.S.X, a junior radiologist with 3-year experience in interpretation of abdominal imaging) on all slices showing the lesion. First, the reader annotated the central area of the lesion and adjacent normal tissue respectively on a transverse, coronal, sagittal section. Then, an approximation algorithm called grow from seeds was used to generate a 3D region of interest (ROI) automatically. Finally, the reader could further optimize ROI manually if necessary.

Before extracting features, each CT scan of each patient was normalized with Z-scores in order to reduce image noise from different CT scanners. Images were then resampled to a voxel size of $1 \times 1 \times 1$ mm^3. With the Radiomics module in 3D Slicer, 851 radiomics features were extracted based on venous phase CT images. After omitting 87 features with missing values, a total of 764 radiomics features were finally obtained.

3. Radiomics features information

A total of 764 radiomics features were extracted based on venous-phase CT images, covering six groups (14 shape features, 16 first-order features, 11 gray level dependence matrix features (GLDM), 12 gray level run length matrix features (GLRLM), 12 gray level size zone matrix features (GLSZM), and 699 wavelet-based features). Detailed information about these features is listed in Table S1.

Feature class	Feature name									
Shape	Elongation	Flatness	LeastAxisLength	MajorAxisLength	Maximum2DDiameterColumn	Maximum2DDiameterRow	Maximum2DDiameterSlice	Maximum3DDiameter	MeshVolume	MinorAxisLength
	Sphericity	SurfaceArea	SurfaceVolumeRatio	VoxelVolume						
First-order	10Percentile	90Percentile	Energy	InterquartileRange	Kurtosis	Maximum	MeanAbsoluteDeviation	Mean	Median	Minimum
	Range	RobustMeanAbsoluteDeviation	RootMeanSquared	Skewness	TotalEnergy	Variance				
GLDM	DependenceEntropy	DependenceNonUniformity	DependenceNonUniformityNormalized	DependenceVariance	GrayLevelNonUniformity	LargeDependenceEmphasis	LargeDependenceHighGrayLevelEmphasis	LargeDependenceLowGrayLevelEmphasis	SmallDependenceEmphasis	SmallDependenceHighGrayLevelEmphasis
	SmallDependenceLowGrayLevelEmphasis									
GLRLM	GrayLevelNonUniformity	LongRunEmphasis	LongRunHighGrayLevelEmphasis	LongRunLowGrayLevelEmphasis	RunEntropy	RunLengthNonUniformity	RunLengthNonUniformityNormalized	RunPercentage	RunVariance	ShortRunEmphasis
	ShortRunEmphasis	ShortRunLowGrayLevelEmphasis								
GLSZM	GrayLevelNonUniformity	LargeAreaEmphasis	LargeAreaHighGrayLevelEmphasis	LargeAreaLowGrayLevelEmphasis	SizeZoneNonUniformity	SizeZoneNonUniformityNormalized	SmallAreaEmphasis	SmallAreaHighGrayLevelEmphasis	SmallAreaLowGrayLevelEmphasis	ZoneEntropy
	ZonePercentage	ZoneVariance								
Wavelet-based	Wavelet transformation based on above features.									

Table S1. List of radiomics features classes. GLDM, gray level dependence matrix; GLRLM, gray level run length matrix; GLSZM, gray level size zone matrix.

Table S2. Detailed information on radiomics features. GLDM, gray level dependence matrix; GLRLM, gray level run length matrix; GLSZM, gray level size zone matrix.

3.Diagnostic criteria of radiological features.

(i) Tumor size: Tumor size was measured on transverse images. The maximum diameter of the tumor was recorded;

(ii) Location: The left margin of the superior mesenteric vein was used to divide pancreatic head/neck and body/tail;

(iii) Lesion contour: Lesion contour was split into two groups named round/ovoid and lobulated. If the lesion contour could not be described as the borders of the same circle, it was defined as a lobulated contour, or it was defined as a round/ovoid contour;

(iv) Wall thickness: If the wall thickness of a cystic lesion was larger than 2 mm, it was considered thick. If the wall was imperceptible or wall thickness smaller than 2 mm, it was considered thin;

(v) Wall enhancement: Wall enhancement was described as line or rim-like enhancement of lesion wall in arterial or venous-phase CT images. The enhanced wall was visible for at least 50% of the lesion circumference;

(vi) Calcification: Calcification presented on the wall or septa was noted as positive;

(vii) Mural nodules: A nodule-like structure on the inner side of the wall or on the septa was noted as mural nodules positive;

(viii) Dilation of the Wirsung duct: The main pancreatic duct was considered dilation if its diameter was larger than 2 mm.

Examples of radiological features are illustrated in Fig S2

Radiological feature	Example	
Tumor size	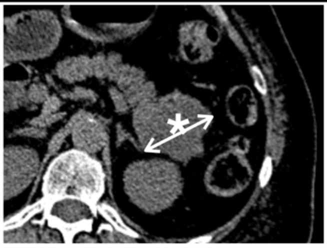	
Location	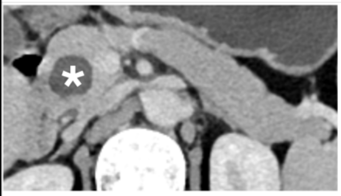<br>Pancreatic head/neck	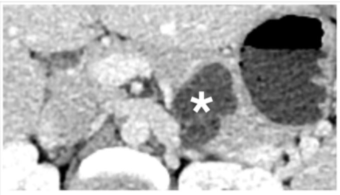<br>Pancreatic body/tail
Lesion contour	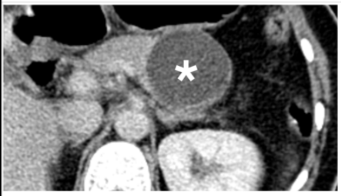<br>Round/oval	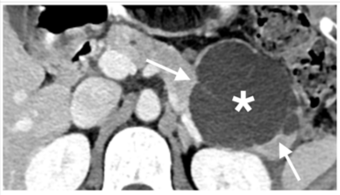<br>Lobulated
Wall thickness	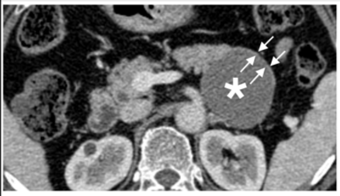<br>Thin	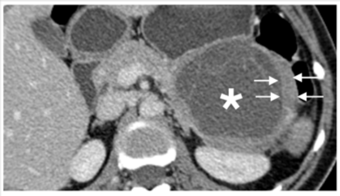<br>Thick
Wall enhancement	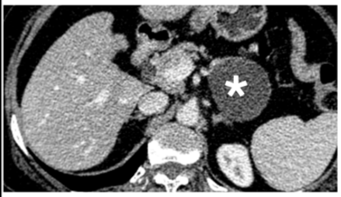<br>Absent	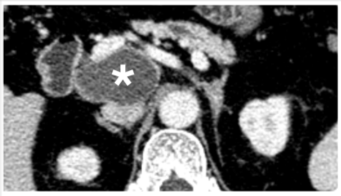<br>Present
Calcification	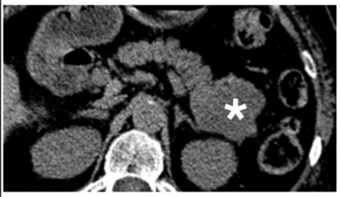<br>Absent	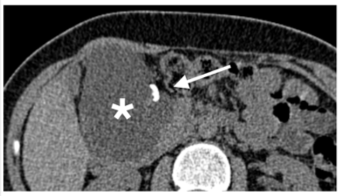<br>Present
Mural nodule	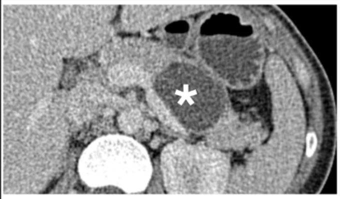<br>Absent	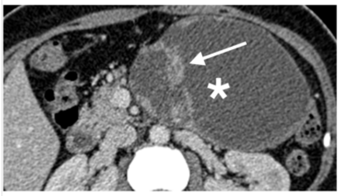<br>Present

Fig S2. Examples of radiological features. An asterisk was used to annotate the target lesion.

4.Reproducibility analysis for radiomics feature extraction.

A total of 764 radiomics features were extracted for each patient. The first step of the feature selection was reproducibility analysis. Thirty patients were randomly chosen to evaluate the inter/intra-observer intraclass correlation coefficient (ICC) of radiomics features. Reader 1 (T.S.X, a junior radiologist with 3-year experience in interpretation of abdominal imaging) repeated segmentation flow twice with time intervals exceeding one week; reader 2 (X.Y.W, a junior radiation oncologist with 4-year experience in irradiation volume segmentation of abdominal tumor) independently performed segmentation flow; the intraclass correlation coefficient (ICC) was calculated to evaluate feature reproducibility. All readers were blinded to the pathological diagnosis during segmentation.

Among all 764 radiomics features, there were 491 and 492 features with inter-observation ICC and intra-observation ICC higher than 0.90, respectively (Fig S3.). Finally, 472 features with both inter- and intra-observation ICC higher than 0.90 were selected for the next step.

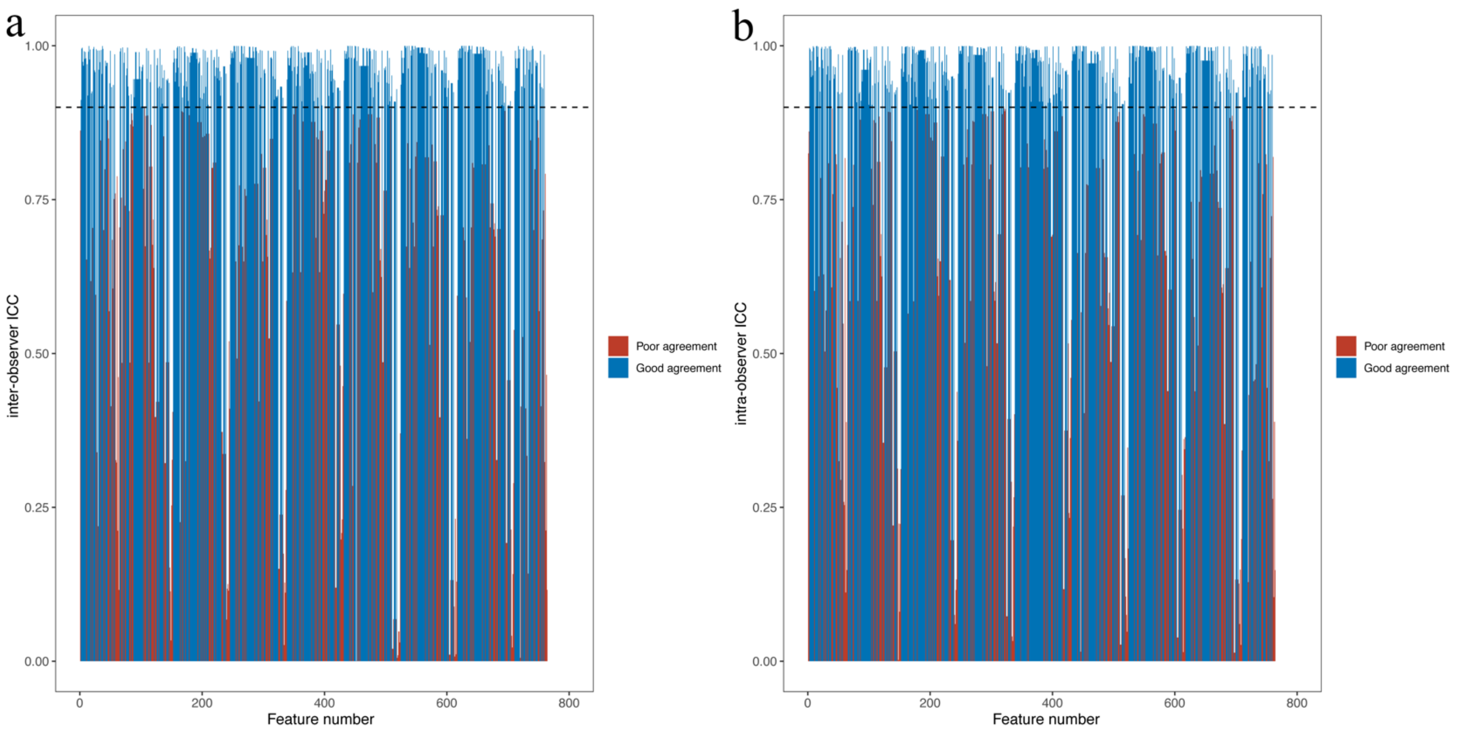

Fig. S3 Reproducibility analysis for radiomics features (a, inter-observer analysis; b, intra-observer analysis).

The x-axis represents the feature number of 764 radiomics features; the y-axis measures features' s ICC. The horizontal dotted line represents the cutoff value of ICC level (0.90). Features with good agreement are annotated by a blue bar, while a poor agreement is annotated by a red bar.

5. R packages in this study

The R packages used in this study were as follows. Random forest algorithm was completed using the 'randomForest' package. The 10-fold cross validation was performed

using the ‘caret’ package. ROC was printed using ‘pROC’ package. The logistic regression model was constrcuted using the ‘rms’ package. The calculation of ICC was done by using the ‘irr’ package.
